# The impact of oral semaglutide on glycemic control and weight reduction: a database analysis of dosing effects in Japanese individuals with type 2 diabetes

**DOI:** 10.3389/fendo.2025.1615516

**Published:** 2025-08-26

**Authors:** Mizuki Ishiguro, Rimei Nishimura

**Affiliations:** Division of Diabetes, Metabolism, and Endocrinology, Department of Internal Medicine, Jikei University School of Medicine, Tokyo, Japan

**Keywords:** GLP-1 receptor agonists, type 2 diabetes, oral antidiabetic drugs, semaglutide, HbA1c

## Abstract

**Objective:**

GLP-1 receptor agonists (GLP-1 RAs) reduce cardiovascular events in type 2 diabetes (T2D), and oral formulations improve accessibility. However, their real-world effectiveness and predictors of response remain unclear. This study assessed the proportions of individuals achieving HbA1c < 7.0% and experiencing ≥3.0% weight reduction after 180 days of maintenance-dose therapy (no dose modification for ≥180 days).

**Methods:**

We retrospectively analyzed 169 participants with T2D (3 mg: n=45; 7 mg: n=92; 14 mg: n=32) treated at a single medical care center in Japan. The cohort included participants with HbA1c ≥ 7.0% at baseline. We evaluated semaglutide changes in HbA1c and weight, and predictors of glycemic response after 180 days of maintenance therapy using logistic regression.

**Results:**

Baseline characteristics included median age 63.0 years, body mass index (BMI) 27.2 kg/m², diabetes duration 10.0 years, and HbA1c 7.7%. Oral semaglutide was initiated as the first or second choice in 45.0% of participants. HbA1c < 7.0% was achieved in 60.0%, 53.3%, and 46.9% of the 3 mg, 7 mg, and 14 mg groups, respectively. Weight reduction ≥ 3.0% occurred in approximately half of participants across all groups. Lower baseline HbA1c (B = -1.330, p < 0.001) and earlier semaglutide use (first/second choice; B = 1.070, p = 0.013) were significant predictors of HbA1c < 7.0%.

**Conclusion:**

Approximately 50% of participants across all dosing groups achieved HbA1c < 7.0% after 180 days of maintenance therapy. Weight reduction (≥ 3.0%) occurred frequently in parallel with HbA1c reduction. Early semaglutide use (first/second choice) and lower baseline HbA1c predicted HbA1c < 7.0% on maintenance therapy.

## 1 Introduction

Globally, the prevalence of diabetes continues to rise, with an estimated 589 million individuals aged 20–79 years (11.0% prevalence) in 2024, according to the IDF Diabetes Atlas, 11^th^ edition (1). This trend is predicted to persist, reflecting similar patterns observed in Japan (2). Diabetes is associated with significant risks for both microvascular (e.g. neuropathy, retinopathy, nephropathy) and macrovascular complications (e.g., cardiovascular disease [CVD] and stroke), which reduce life expectancy and place a significant burden on healthcare systems (3, 4). Consequently, achieving HbA1c levels below 7.0% has been established as a key therapeutic goal to mitigate the risk of complications (5). Early achievement of glycemic control following diagnosis has been demonstrated to mitigate the risk of onset and progression of macrovascular complications, a phenomenon known as the “legacy effect,” and improve long-term clinical outcomes (6). Moreover, maintaining diabetes remission, even in advanced stages, has been associated with a reduction in the progression of chronic kidney disease (CKD) and CVD (7).

Since 2022, the American Diabetes Association (ADA) has advocated for the intensive use of SGLT2 inhibitors and GLP-1 RAs in individuals with CKD (8). This recommendation is grounded in robust evidence derived from meta-analyses and large-scale clinical trials, which have demonstrated the efficacy of these drugs in the attenuation of CKD progression (9–21).

In Japan, GLP-1 RAs have been available since 2010, with liraglutide and exenatide as the initial agents. Subsequent advancements led to the introduction of weekly injectable formulations, including exenatide in 2013, dulaglutide in 2015, and semaglutide in 2020. In 2021, oral semaglutide was introduced, utilizing SNAC (salcaprozate sodium) technology to enhance gastrointestinal absorption. Given the aversion to injectable therapies among certain populations in Asia, oral formulations have been rapidly adopted. Although both oral and injectable formulations demonstrate comparable efficacy at equivalent plasma concentrations, oral formulations exhibit higher interindividual variability (22). Despite the increasing use of oral semaglutide, real-world evidence regarding its efficacy and the predictors of its effectiveness remains limited. Therefore, the aim of this research is to provide predictors of treatment response in clinical practice for oral GLP-1 RA.

## 2 Methods

### 2.1 Study subjects

This study included participants with T2D who had an HbA1c level of >7.0% and were initiated on oral semaglutide therapy at Jikei University Hospital between January 2022 and December 2023. Data were collected using the electronic medical record (EMR) system, which facilitates various factors, including age, gender, test results, diagnoses, and medication details, all of which are coded according to the ICD-10 classification system. Physical findings such as height, weight, and blood pressure were also recorded through the EMR system.

The study targeted participants with T2D who had been continuously prescribed oral semaglutide at doses of 3 mg, 7 mg, or 14 mg for a minimum of 180 days, with the first prescription of each dose serving as the baseline date. The exclusion criteria were:(1) participants with type 1 diabetes (T1D) or pregnancy, (2) participants aged < 20 years, (3) participants who had already been prescribed oral semaglutide during the initial consultation, (4) participants who were newly prescribed or had experienced dose escalation of oral semaglutide during hospitalization, (5) participants whose other oral antidiabetic drugs were changed within 180 days after initiation of 3 mg, 7 mg, or 14 mg maintenance doses of oral semaglutide, respectively, (6) participants who switched from non-incretin oral antidiabetic drugs to oral semaglutide, or (7) participants who transitioned from injectable GLP-1 RAs to oral semaglutide. The specific explanation for exclusion criteria were as follows: (3) uncertainty regarding the timing of the initial prescription of oral semaglutide, (4) rapid dose escalation of oral semaglutide during hospitalization, (5) potential confounding effects on HbA1c levels, and (6, 7) difficulties in accurately assessing the therapeutic effects of oral semaglutide.

### 2.2 Measurements

For the study population, data were collected at the time of the initial prescription of 3 mg oral semaglutide, including age, duration of diabetes, weight, body mass index (BMI), systolic and diastolic blood pressure, and laboratory test results (e.g., AST, ALT, γGTP, urea nitrogen [UN], creatinine [Cr], estimated glomerular filtration rate [eGFR], uric acid [UA], triglycerides, LDL cholesterol [LDL-C], HDL cholesterol [HDL-C], postprandial plasma glucose, HbA1c, and complete blood counts). Data on concomitant oral antidiabetic drugs were also recorded.

Participants were categorized according to the line of therapy at which oral semaglutide was initiated. Those who received oral semaglutide as the first- or second-choice therapy were defined as the “first- or second-choice therapy” group, whereas those who received it as the third or subsequent choice were classified as the “third- or subsequent-choice therapy” group.

The median number of outpatient visits within 180 days after the initiation of oral semaglutide maintenance dose was two, defined as the “1st visit” and “2nd visit.” Additionally, the first outpatient visit occurring after the 180-day period following the initiation of the maintenance dose was defined as the “first visit after 180 days.” These visits were used to monitor clinical parameters, including HbA1c and body weight, with follow-up continued until the first visit after 180 days. Outpatient follow-up visits were not standardized and were scheduled at the discretion of the treating physician, typically occurring at two to three months.

### 2.3 Primary outcomes

The primary outcomes included changes in the mean HbA1c levels up to the first visit after 180 days with oral semaglutide at doses of 3mg, 7mg, or 14mg, the proportion of individuals achieving an HbA1c < 7.0% (The proportion of individuals achieving HbA1c < 7.0%), and the proportion of individuals achieving a ≥ 3.0% reduction in body weight from the initiation of 3 mg oral semaglutide (The proportion of individuals achieving a weight reduction ≥ 3.0%).

### 2.4 Secondary outcomes

Secondary outcomes included the preference for oral semaglutide among oral antidiabetic drugs and the identification of predictive factors associated with the achievement of HbA1c < 7.0%.

### 2.5 Statistical analysis

All participants were stratified into three groups based on the maintenance dose of semaglutide. Differences in baseline characteristics among the groups were assessed using the Kruskal-Wallis test for continuous variables and the chi-square test for categorical variables. Variables that showed significant differences in the Kruskal-Wallis test were further examined using Bonferroni correction for multiple comparisons. Logistic regression analysis with forced entry was performed to identify factors associated with the achievement of HbA1c < 7.0%. Statistical analyses were conducted using SPSS Version 29.0 (SPSS Inc., Chicago, IL, USA). Data are presented as medians [interquartile ranges] and percentages (%), with statistical significance set at p < 0.05.

## 3 Results

### 3.1 Participant background

#### 3.1.1 Study population

**Figure 1:**
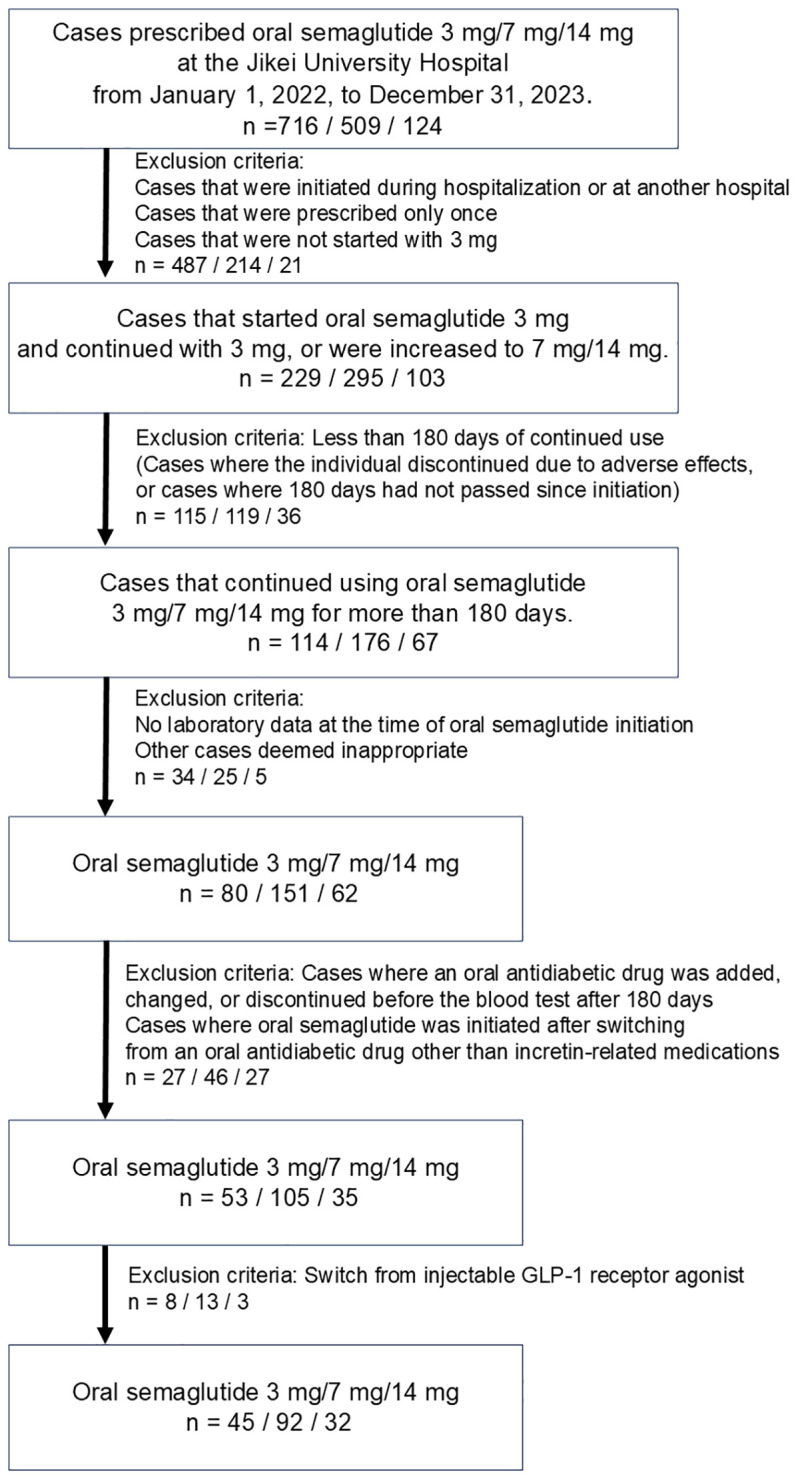
Criteria for selection of study population. This figure illustrates the criteria used to select participants for this study, including exclusion based on factors such as initial dose, treatment duration, and availability of baseline data.

Between 2022 and 2023, a total of 1,349 participants with T2D were prescribed oral semaglutide. The cohort was categorized into three dosage groups: 3 mg (n = 716), 7 mg (n = 509), and 14 mg (n = 124). Of these, 1,180 participants were excluded for the following reasons: initiation during hospitalization, initiation at another institution, having received only a single prescription for oral semaglutide, initiation at a dose other than 3 mg, treatment duration of less than 180 days, missing baseline laboratory data, changes in concomitant oral antidiabetic drugs, or prior use of injectable GLP-1 RAs. Following the application of inclusion criteria, such as maintaining the prescribed dosage for a minimum of 180 days, 169 participants were included in the final analysis. This cohort comprised 45 participants in the 3 mg group, 92 participants in the 7 mg group, and 32 participants in the 14 mg group, respectively (**Figure 1**).

**Figure 2:**
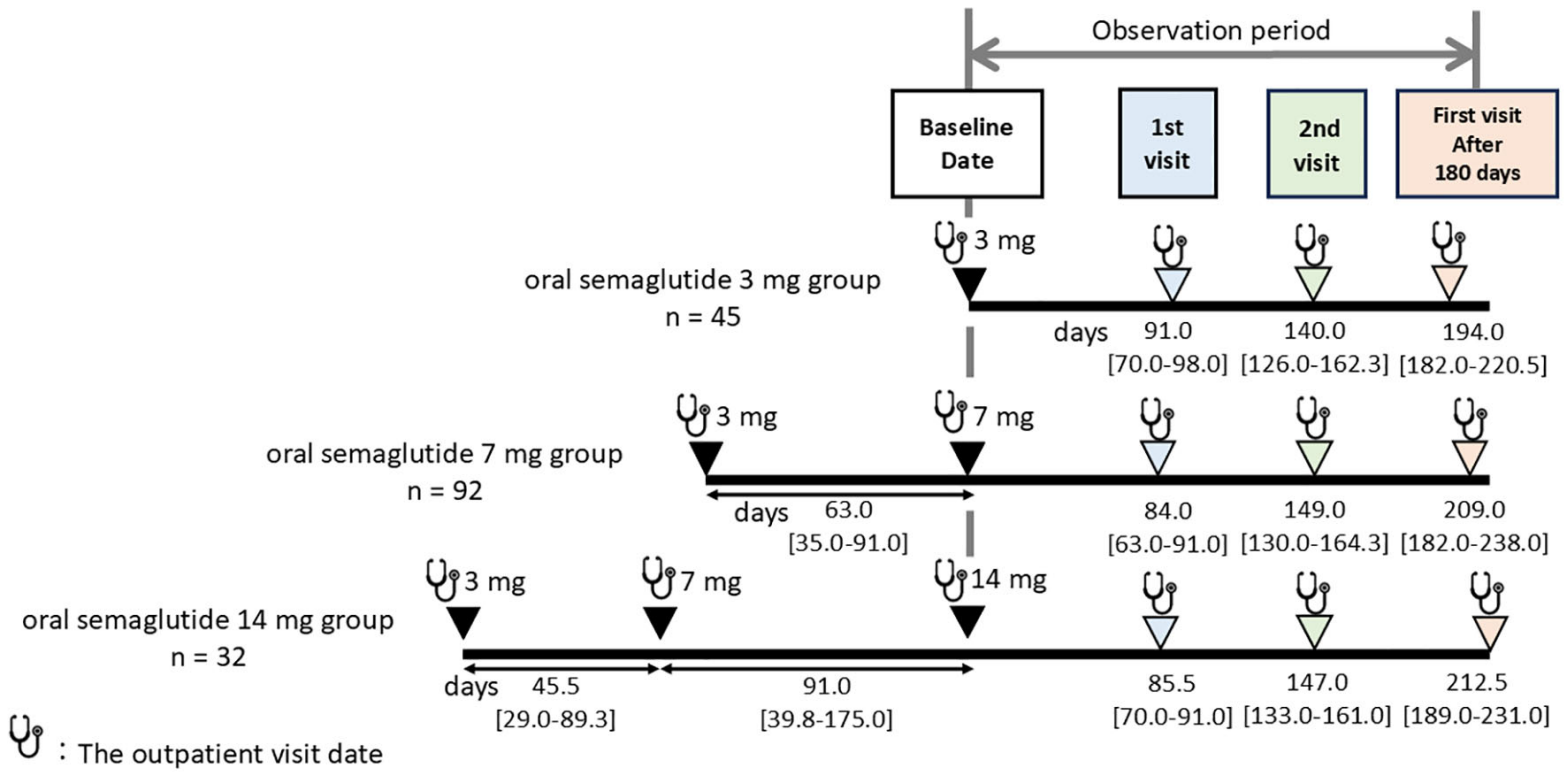
Median number of days to blood tests and weight measurements across different oral semaglutide maintenance doses. The median number of outpatient visits within the 180-day period after starting maintenance dose (3 mg, 7 mg, or 14 mg) of oral semaglutide was two, defined as the “1st visit” and “2nd visit.” Additionally, the first outpatient visit occurring after the 180-day period was designated as the “first visit after 180 days.” Follow-up times are presented as median [interquartile range] in days.

The median number of outpatient visits within the 180-day period was two (the “1st visit” and “2nd visit”, as defined in Methods.) Additionally, the interval until the first visit after 180 days was analyzed, and changes in data at these visits were subsequently compared across the dosage groups. The median number of days to the 1st visit, 2nd visit, and the first visit after 180 days were 84.0 [69.0–91.0], 147.0 [131.0–161.0], and 203.0 [182.0–231.0], respectively, with no statistically significant differences observed among the three groups (p = 0.315, p = 0.653, p = 0.182) (**Figure 2**).

#### 3.1.2 Baseline characteristics of the study population

(**Table 1**) The study population consisted of 119 males (70.4%) and 50 females (29.6%), with a median age of 63.0 years. The median values for height, weight, and BMI were 166.0 cm, 75.0 kg, and 27.2 kg/m², respectively. The cohort had a median diabetes duration of 10.0 years, with a baseline median HbA1c of 7.7%. Of the total cohort, 117 participants (69.2%) had transitioned from DPP-4 inhibitors, and 5 participants (3.0%) were receiving concurrent insulin therapy.

**Table 1:** Baseline characteristics of the participants.

	All	3mg	7mg	14mg	P value†
Participant evaluated (N)	169	45	92	32	
Sex (Males/Females, N [%])	119 (70.4)/50 (29.6)	28 (62.2)/17 (37.8)	63 (68.5)/29 (31.5)	28 (87.5)/4 (12.5)	0.047^*^
Prior administration of DPP-4 inhibitor, N (%)	117 (69.2)	22 (48.9)	74 (80.4)	21 (65.6)	<0.001^*^
Concomitant use of insulin, N (%)	5 (3.0)	2 (4.4)	2 (2.2)	1 (3.1)	0.761
Age (years)	63.0 [54.0-70.0]	65.0 [57.0-72.5]	63.0 [55.0-69.0]	56.5 [47.3-67.8]	0.085
Body height (cm)	166.0 [160.0-171.0]	164.5 [158.0-171.0]	165.5 [160.0-170.8]	168.0 [164.3-173.8]	0.189
Body weight (kg)	75.0 [66.0-87.4]	72.9 [66.8-85.8]	74.0 [65.3-83.8]	82.0 [67.0-91.0]	0.152
BMI (kg/m^2^)	27.2 [24.4-30.9]	26.5 [23.9-33.6]	27.4 [24.2-30.4]	27.5 [26.2-32.2]	0.320
Duration of diabetes (years)	10.0 [5.0-17.0]	8.0 [4.5-21.0]	11.0 [6.0-17.0]	9.0 [6.0-13.5]	0.712
SBP (mmHg)	130.0 [121.5-139.5]	130.0 [122.0-142.0]	128.0 [120.0-138.0]	132.0 [124.0-140.3]	0.425
DBP (mmHg)	80.0 [72.0-87.0]	80.0 [72.0-87.0]	80.0 [71.0-87.3]	80.0 [75.0-87.5]	0.773
AST (U/L)	23.0 [18.0-37.0]	24.0 [18.0-31.0]	23.5 [18.0-39.3]	22.5 [17.3-38.3]	0.889
ALT (U/L)	28.0 [17.0-51.5]	25.0 [16.0-46.0]	29.0 [18.3-54.5]	30.0 [17.3-52.0]	0.476
γGTP (U/L)	37.0 [25.0-69.8]	27.0 [22.0-65.3]	38.5 [27.8-73.3]	49.0 [26.0-73.8]	0.131
UN (mg/dL)	17.0 [14.0-21.0]	18.0 [14.0-23.0]	16.0 [14.0-20.0]	18.5 [13.0-20.8]	0.488
Cr (mg/dL)	0.85 [0.66-1.04]	0.90 [0.71-1.16]	0.80 [0.62-0.96]	0.85 [0.67-1.07]	0.122
eGFR (ml/min/1.73m^2^)	68.5 [54.0-85.8]	61.0 [43.5-83.5]	70.0 [58.0-86.0]	73.5 [52.5-95.3]	0.062
UA (mg/dL)	5.6 [4.6-6.7]	5.6 [5.0-6.8]	5.5 [4.4-6.6]	6.1 [5.1-6.7]	0.258
TG (mg/dL)	150.5 [101.0-233.8]	166.0 [94.5-221.0]	144.0 [98.0-206.0]	179.0 [114.0-361.5]	0.166
HDL (mg/dL)	53.0 [47.0-64.0]	52.0 [44.5-64.3]	55.0 [47.5-64.0]	50.0 [44.3-61.8]	0.317
LDL (mg/dL)	108.0 [86.0-125.5]	108.5 [89.0-132.8]	103.5 [80.5-122.0]	118.0 [97.0-127.0]	0.204
WBC (/μL)	6700 [5600–8050]	6700 [5600-7850]	6700 [5500-8000]	6650 [5750-8325]	0.895
RBC (×10^4^/μL)	499 [475-535]	494 [461-518]	493 [472-541]	525 [500-545]	0.016^*^
Hb (g/dL)	14.9 [14.2-15.9]	14.7 [13.9-15.5]	14.8 [14.1-15.7]	15.5 [14.8-16.4]	0.026^*^
Ht (%)	45.7 [43.2-48.6]	45.1 [42.6-47.5]	45.6 [43.0-48.0]	47.8 [45.6-49.3]	0.021^*^
Plt (×10^4^/μL)	21.2 [18.4-25.7]	21.5 [19.2-25.1]	20.8 [18.0-25.7]	19.9 [17.1-25.9]	0.506
PPG (mg/dL)	147.0 [123.0-175.5]	145.0 [125.5-169.0]	148.0 [119.3-172.0]	159.0 [131.3-194.3]	0.331
HbA1c (%)	7.7 [7.2-8.3]	7.6 [7.0-8.2]	7.7 [7.3-8.2]	8.1 [7.3-8.7]	0.081

BMI, body mass index; SBP systolic blood pressure; DBP diastolic blood pressure; eGFR, estimated glomerular filtration rate as calculated by use of the formula: men (mL/min/1.73m^2^) =^194×Cr-1.094^×age^-0.287^; women(mL/min/1.73m^2^) =194×Cr^-1.094^×age^-0.287^×0.739; TG, triglycerides; LDL-C, low-density lipoprotein cholesterol; HDL-C, high-density lipoprotein cholesterol; PPG, postprandial plasma glucose.

Categorical variables are shown as numbers and percentages (%), and were compared using the chi-square test. Fisher’s exact test was used when expected cell counts were <5 (e.g., concomitant insulin use). Categorical variables with significant differences among dose groups (i.e., sex and prior use of DPP-4 inhibitors) are also illustrated as bar charts in **Supplementary Figure 1**.

Continuous variables are presented as medians [interquartile range] and were compared using the Kruskal–Wallis test. For variables with significant differences in the Kruskal–Wallis test, pairwise comparisons among dose groups (3 mg, 7 mg, and 14 mg) were performed using Bonferroni correction (significance threshold set at p < 0.016).

Significant *post-hoc* differences (Bonferroni-adjusted) were observed as follows: RBC (3mg vs. 14mg, p = 0.014), Hb (p = 0.027), Ht (p = 0.017). Box plots for hematological parameters with significant differences (RBC, Hb, Ht) are provided in **Supplementary Figure 2**.

†P-values indicate comparisons among oral semaglutide dose groups. A p-value < 0.05 was considered statistically significant unless otherwise noted.

The symbol '*' indicates statistical significance at p < 0.05.

Comparison of baseline characteristics across dosage groups (3 mg, 7 mg, and 14 mg) revealed a significantly higher proportion of men in the 14mg group compared to the 3mg group (p=0.047). Additionally, a significantly higher proportion of participants transitioned from DPP-4 inhibitors was observed in the 7 mg group compared to the 3 mg group (p < 0.001). These findings are illustrated in **Supplementary Figure 1**.

With respect to hematological parameters, Bonferroni-adjusted pairwise comparisons showed that red blood cell (RBC) count, hemoglobin (Hb), and hematocrit (Ht) levels were significantly higher in the 14 mg group than in the 3 mg group (p = 0.014, p = 0.027, and p = 0.017, respectively), as shown in **Supplementary Figure 2**.

### 3.2 Preference for oral semaglutide and concomitant antidiabetic drugs

**Figure 3:**
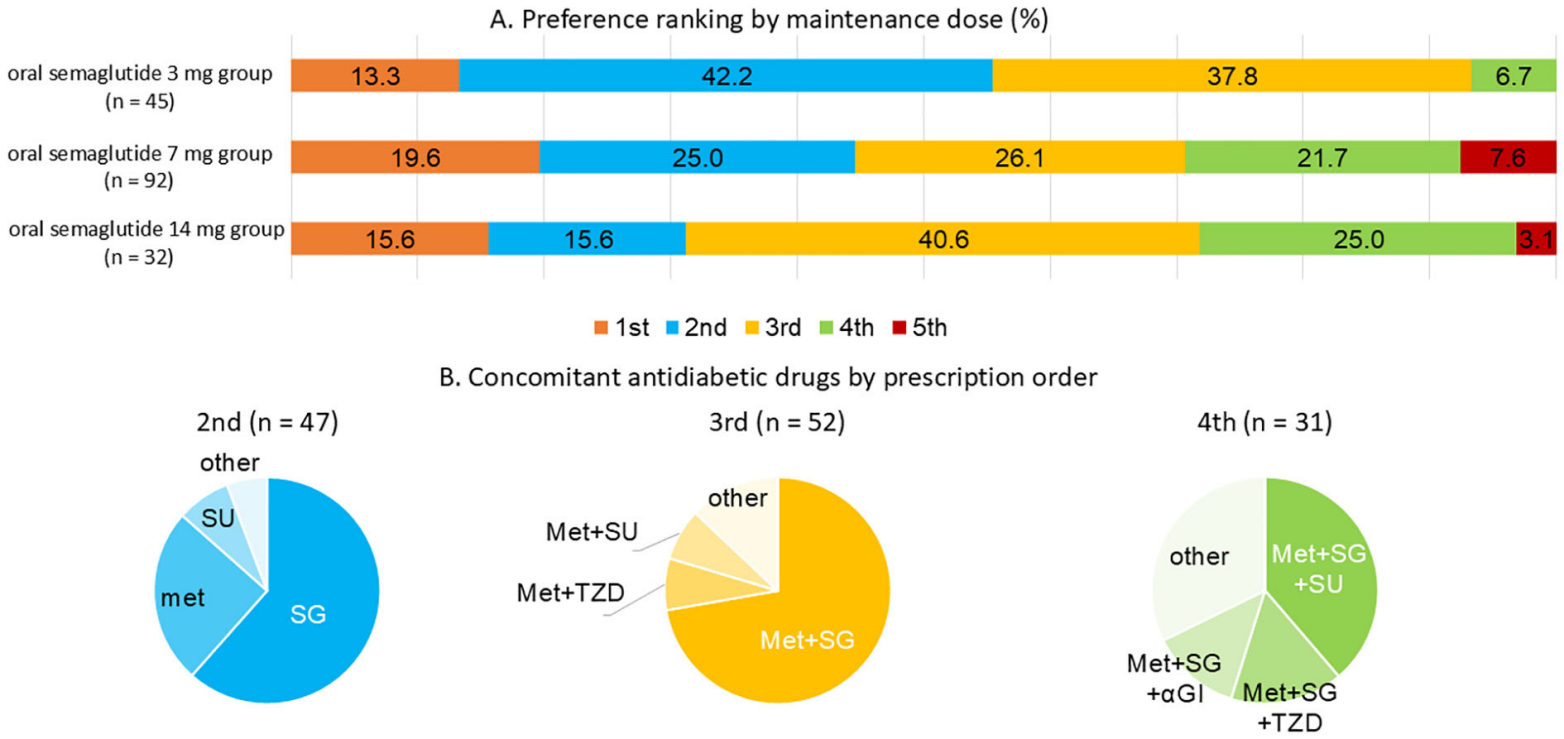
**(A)** Distribution of oral semaglutide preference by maintenance dosage. **(B)** Concomitant oral antidiabetic drugs used according to prescription ranking of oral semaglutide. In the figure, therapy rankings are abbreviated as follows: 1st = the first-choice therapy; 2nd = the second-choice therapy; 3rd = the third-choice therapy; 4th = the fourth-choice therapy; 5th = the fifth-choice therapy. Panel **(A)** shows the proportion of individuals receiving oral semaglutide as 1st, 2nd, 3rd, 4th, or 5th choice therapy by maintenance dose group (3 mg, 7 mg, or 14 mg). Panel **(B)** summarizes the concomitant oral antidiabetic drugs prescribed within each therapy choice group from 2nd to 4th choice. SG refers to SGLT2 inhibitors, Met to metformin, and TZD to thiazolidinediones. Number of participants by therapy choice and dose group are as follows: 1st: 3 mg (n = 6), 7 mg (n = 18), 14 mg (n = 5); 2nd: 3 mg (n = 19), 7 mg (n = 23), 14 mg (n = 5); 3rd: 3 mg (n = 17), 7 mg (n = 24), 14 mg (n = 13); 4th: 3 mg (n = 3), 7 mg (n = 20), 14 mg (n = 8).

(**Figure 3**) The preferences for drug selection at the initiation of oral semaglutide 3 mg were analyzed by dosage group (**Figure 3A**). The overall distribution of selection preferences was as follows: the first-choice therapy: 17.2%, the second-choice therapy: 27.8%, the third-choice therapy: 32.0%, the fourth-choice therapy: 18.3%, and the fifth-choice therapy: 4.7%.

At the initiation of oral semaglutide 3 mg, participants in the higher-dose groups, particularly those in the 14 mg group, were more likely to have been receiving two or three other oral antidiabetic drugs prior to initiation. In cases where participants were switched from DPP-4 inhibitor monotherapy, semaglutide was defined as the first-choice therapy.

Selection preferences for concomitant oral antidiabetic drugs were also analyzed, and their distribution across the second- to fourth-choice therapies is illustrated in **Figure 3B**. When semaglutide was prescribed as the second-choice therapy, metformin or SGLT2 inhibitors were commonly used. In contrast, when it was prescribed as the third- or subsequent-choice therapy, the combination of metformin and SGLT2 inhibitors was commonly observed.

### 3.3 Impact on glycemic control and weight

**Figure 4:**
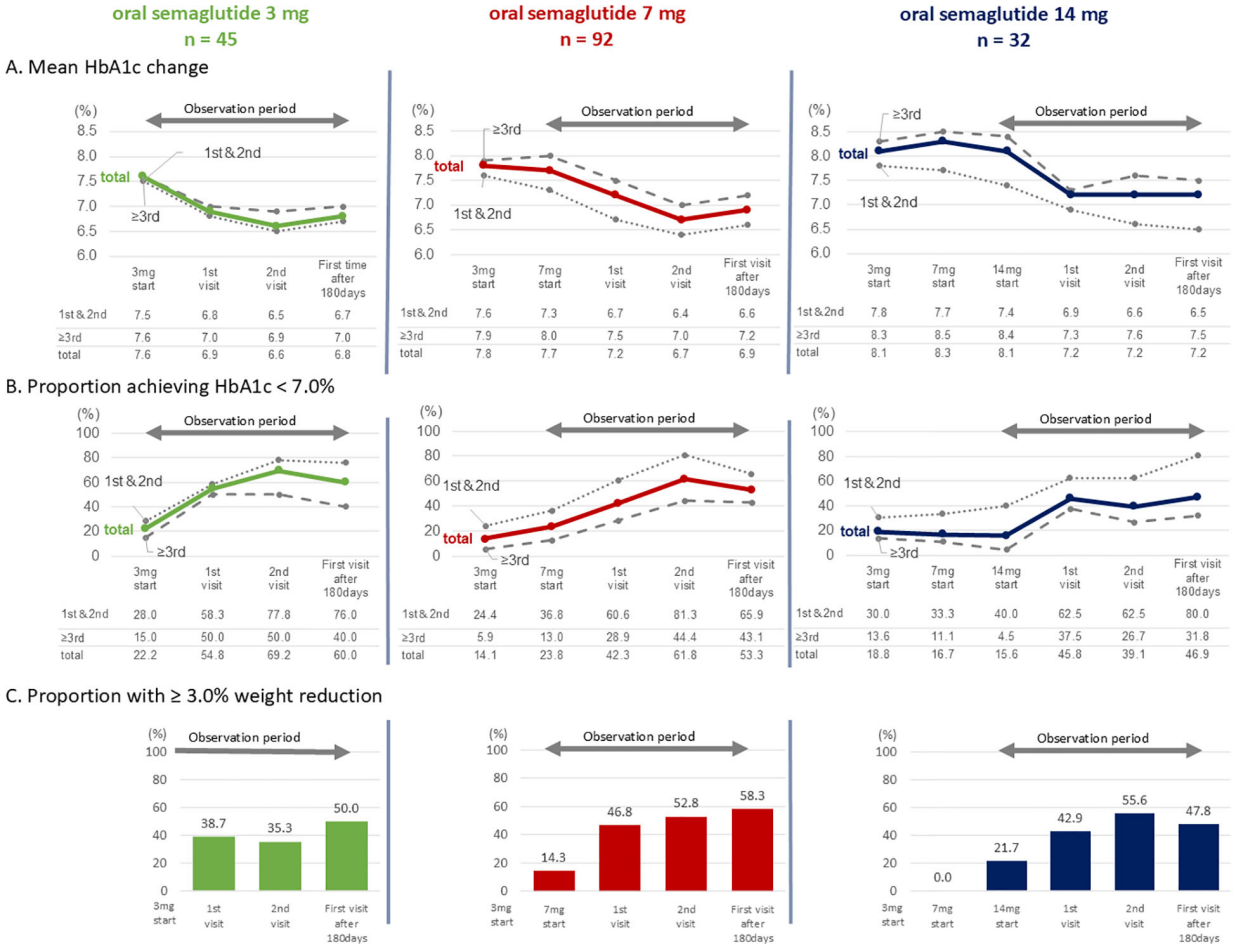
**(A)** Mean changes in HbA1c; **(B)** Proportion of individuals achieving HbA1c < 7.0%; **(C)** Proportion of individuals achieving a weight reduction ≥ 3.0%. Each parameter is stratified by oral semaglutide dose groups. In **(A, B)** groups in which semaglutide was prescribed as the first- or second-choice therapy (abbreviated as “1st & 2nd” in the figure) are shown by dotted lines, the third- or subsequent-choice therapy groups (abbreviated as “≥3rd”) by dashed lines, and the overall cohort (abbreviated as “total”) by solid lines.

(**Figure 4**) The proportion of individuals achieving HbA1c < 7.0% was analyzed according to dosage group (**Figure 4B**). In the 3 mg group, 60.0% of individuals had HbA1c < 7.0% after 180 days. When stratified by selection preference, the proportions of individuals achieving HbA1c < 7.0% were 76.0% for the first- or second-choice therapy and 40.0% for the third- or subsequent-choice therapy (**Figure 4B**; oral semaglutide 3mg group).

In the 7 mg group, the proportion of individuals achieving HbA1c < 7.0% after 180 days was 53.3%. When divided by selection preference, the proportion of individuals achieving HbA1c < 7.0% were 65.9% for the first- or second-choice therapy and 43.1% for the third- or subsequent-choice therapy (**Figure 4B**; oral semaglutide 7mg group).

In the 14 mg group, the proportion of individuals achieving HbA1c < 7.0% after 180 days was 46.9%. The corresponding proportions were 80.0% for the first- or second-choice therapy and 31.8% for the third- or subsequent-choice therapy, respectively (**Figure 4B**; oral semaglutide 14 mg group).

The proportion of individuals achieving a weight reduction ≥3.0% was also analyzed (**Figure 4C**). After 180 days, this proportion was 50.0% in the 3 mg group, 58.3% in the 7 mg group, and 47.8% in the 14 mg group, respectively.

### 3.4 Factors predicting achievement of HbA1c < 7.0%

(**Table 2**) Logistic regression analysis was conducted to identify predictors of achieving HbA1c < 7.0% at the first visit after 180 days. The results indicated that for every 1% increase in baseline HbA1c, the odds of achieving HbA1c < 7.0% decreased (OR = 0.264, 95% CI: 0.144-0.486, p < 0.001). Moreover, when oral semaglutide was selected as the first- or second-choice therapy, the odds of achieving HbA1c < 7.0% were 2.917 (95% CI: 1.249-6.813, p=0.013) compared to its use as the third- or subsequent-choice therapy.

**Table 2:** Factors predicting achievement of HbA1c < 7.0%.

Independent variables	Multivariate logistic regression analysis					
	B (S.E.)	Wald	Odds ratio	95% confidence interval		P value†
				Lower limit	Upper limit	
Age (years)	0.003 (0.024)	0.019	1.003	0.957	1.052	0.890
Sex †	0.151 (0.501)	0.091	1.163	0.436	3.107	0.763
BMI (kg/m^2^)	-0.038 (0.052)	0.541	0.962	0.869	1.066	0.462
AST (U/L)	0.002 (0.009)	0.062	1.002	0.985	1.020	0.803
eGFR (ml/min/1.73m^2^)	0.006 (0.010)	0.398	1.006	0.987	1.026	0.528
Hb (g/dL)	-0.060 (0.173)	0.120	0.942	0.671	1.323	0.729
HbA1c (%)	-1.330 (0.311)	18.313	0.264	0.144	0.486	<0.001^*^
The use of oral semaglutide 7mg ‡	-0.297 (0.498)	0.355	0.743	0.280	1.973	0.551
The use of oral semaglutide 14mg §	0.421 (0.658)	0.409	1.523	0.420	5.527	0.522
Prescription Rank (1st/2nd vs 3rd or subsequent) ¶	1.070 (0.433)	6.114	2.917	1.249	6.813	0.013^*^

The achievement of HbA1c < 7.0% was coded as 1, and non-achievement as 0. A logistic regression analysis was performed using the forced entry method. The coding of variables was as follows:

†. Males were coded as 0 and females as 1.

‡. The use of oral semaglutide 7 mg was coded as 1, while 3 mg and 14 mg were coded as 0.

§. The use of oral semaglutide 14 mg was coded as 1, while 3 mg and 7 mg were coded as 0.

¶. Prescriptions ranked as the first or second choice were coded as 1, and whereas those ranked third or subsequent were coded as 0.

'*' indicates statistical significance at p < 0.05.

## 4 Discussion

This study examined the proportion of individuals achieving HbA1c < 7.0% and ≥ a 3.0% weight reduction following continued use of oral semaglutide. One notable study on the efficacy of oral semaglutide in Japanese individuals with T2D is the PIONEER 9 study (23). In this study, the effects of oral semaglutide monotherapy following dietary therapy or a switch from one oral antidiabetic drug were evaluated. The proportion of individuals achieving HbA1c < 7.0% after 26 weeks of oral semaglutide administration were reported as 55.8%, 73.3%, and 79.5% for the 3 mg, 7 mg, and 14 mg groups, respectively. Additionally, a sub-analysis of the PIONEER 3 study highlighted the proportion of individuals achieving HbA1c < 7.0% in Japanese individuals treated with metformin alone or in combination with sulfonylureas. The proportion of individuals with HbA1c < 7.0% after 26 weeks of oral semaglutide treatment at 3 mg, 7 mg, and 14 mg was 26.7%, 43.8%, and 56.4%, respectively (24). In PIONEER3, the proportion of individuals achieving HbA1c < 7.0% was lower compared to PIONEER 9. Which may be attributed to the poor baseline HbA1c levels in the target population, despite the use of metformin or a combination of metformin and sulfonylureas. In this study, the proportion of individuals achieving HbA1c < 7.0% for oral semaglutide as the first- or second-choice therapy after 180 days was 76.0%, 65.9%, and 80.0% for the 3 mg, 7 mg, and 14 mg groups, respectively, which is comparable to the proportion reported in the PIONEER 9 study. The higher achievement proportion observed in the 3 mg group compared to previous reports may be attributed to lower baseline HbA1c levels.

As demonstrated in this study, GLP-1 RAs are rarely used as monotherapy in real-world clinical practice. When oral semaglutide is prescribed as the second- or third-choice therapy, it is often combined with metformin and SGLT2 inhibitors. Therefore, in this study, we examined the proportion of individuals achieving of HbA1c < 7.0%, including those who used oral semaglutide in combination with other antidiabetic drugs. Regarding weight reduction, we referred to the goal for Japanese individuals with obesity, which is a weight reduction of at least 3.0% of current body weight, and analyzed the proportion of individuals achieving a weight reduction ≥3.0% following the initiation of 3mg oral semaglutide.

In contrast to the results obtained when oral semaglutide was used as the first- or second-choice therapy, which were comparable to those reported in PIONEER 9 study, its use as the third- or subsequent-choice therapy led to a significant decrease in the proportion of individuals achieving HbA1c < 7.0%. Specially, the proportions were 40.0%, 43.1%, and 31.8% for the 3 mg, 7 mg, and 14 mg dose groups, respectively. The lower achievement rates may be attributed to the effects of concomitant antidiabetic drugs, as demonstrated in PIONEER 3 study, as well as the potential presence of insulin resistance resulting from chronic poor glycemic control. As shown in **Table 2**, the efficacy of oral semaglutide appears to be limited in individuals who have already used multiple prior oral antidiabetic drugs. On the other hand, for individuals with T2D who may face challenges in treatment, it is advisable to consider introducing oral semaglutide early the treatment course, ideally by the second-choice therapy. In the 14 mg group, there was only a minimal change in the proportion of individuals achieving HbA1c <7.0% between the initiation of 3 mg and the escalation to 7 mg or 14 mg oral semaglutide. However, the proportion of individuals achieving HbA1c < 7.0% showed an increasing trend with continued use of 14 mg oral semaglutide. A similar trend was noted in the 7 mg group, and logistic regression analysis indicated that the use of 7 mg or 14 mg oral semaglutide was not a significant predictor of achieving HbA1c <7.0% (**Table 2**). These findings suggest that if glycemic control goals are not achieved with oral semaglutide, dosage escalation, with continued use for at least 90 days (equivalent to the 1st visit in this study), should be considered, provided it is tolerated.

Furthermore, in all groups, an increase in the proportion of individuals with a weight reduction ≥ 3.0% was observed alongside the increase in the proportion of individuals achieving HbA1c < 7.0%. These findings suggest a potential association between weight reduction and achieving HbA1c < 7.0%, however, the casual relationship remains to be elucidated. However, as shown in **Table 2**, continuing 14 mg oral semaglutide does not necessarily guarantee achievement of HbA1c < 7.0%, with approximately half of the individuals not achieving the desired outcome.

In this study, significant differences were observed across dosage groups, including a higher proportion of male participants in the 14 mg group and a greater number of participants switched from DPP-4 inhibitors in the 7 mg group (**Supplementary Figure 1**). These baseline differences may have influenced both the selection of oral semaglutide dosage and subsequent treatment outcomes. Participants switching from DPP-4 inhibitors may have required higher doses due to inadequate glycemic control with prior therapy. Although sleep apnea syndrome (SAS) was not assessed in this study, the elevated red blood cell (RBC) count, hemoglobin (Hb), and hematocrit (Ht) levels observed in the 14 mg group may be attributed to hypervolemia. Furthermore, both obesity and male sex have been independently associated with elevated hematological parameters (25, 26). In the present study, the 14 mg group had a significantly higher proportion of male participants and tended to have a higher BMI compared to other groups, which may have contributed to the elevations in RBC, Hb, and Ht observed (**Supplementary Figure 2**). Therefore, such background factors should be considered when interpreting the treatment effects across dosage groups.

This study has several limitations. First, the study was conducted at a single institution (a university hospital), with a small sample size, and was limited to facilities accredited by the Japan Diabetes Society, which raises concerns about the generalizability of the findings. Moreover, outpatient follow-up intervals were not standardized and typically ranged from two to three months, depending on the discretion of the treating physician. In other clinical settings, particularly those with more frequent follow-up visits after treatment initiation, the timing and assessment of treatment discontinuation or adverse events may differ. Additionally, the small sample size precluded a stratified analysis of weight reduction by selection preference. This study also focused exclusively on Japanese individuals, preventing comparisons of glycemic variability across different ethnicities. Finally, the relatively short duration of the study did not allow for an analysis of diabetic complications. In addition, for laboratory parameters other than HbA1c and body weight, only baseline data were available. Participants who experienced changes in concomitant oral antidiabetic drugs during the observation period were excluded, which precluded assessment of the impact of such changes. These limitations should be considered when interpreting the results.

Despite these limitations, this study offers valuable insights into the prescribing trends of oral semaglutide in real-world clinical practice, variations in the proportion of individuals achieving HbA1c < 7.0% according to selection preference, and the proportion of individuals achieving a weight reduction ≥ 3.0%.

## 5 Conclusions

Among participants who continued oral semaglutide at doses of 3 mg, 7 mg, or 14 mg for 180 days without dosage adjustments, the proportion of individuals achieving HbA1c < 7.0% was 60.0%, 53.3%, and 46.9%, respectively. When oral semaglutide was used as the first- or second-choice therapy, the proportion of individuals achieving HbA1c < 7.0% was higher compared to its use as the third- or subsequent-choice therapy. Along with the increase in the proportion of individuals achieving HbA1c < 7.0%, the proportion of those who experienced a weight reduction ≥3.0% also increased. Lower baseline HbA1c levels and the use of oral semaglutide as the first- or second-choice therapy were identified as predictors for achieving HbA1c < 7.0%.

## Data Availability

The data analyzed in this study is subject to the following licenses/restrictions: The dataset is restricted due to privacy and ethical considerations. Access is limited to authorized researchers and requires approval from the institutional review board (IRB) at Jikei University Hospital. The data cannot be publicly shared to ensure the protection of patient confidentiality and comply with data protection laws. Requests to access these datasets should be directed to Requests to access the dataset can be made through the official website of Jikei University Hospital: https://www.hosp.jikei.ac.jp/.

## Supplementary material

Supplementary Figure 1 Bar charts of categorical baseline characteristics with significant differences across oral semaglutide dose groups. **(A)** Proportion of male participants. **(B)** Proportion of participants with prior use of DPP-4 inhibitors. A significantly higher proportion of males was observed in the 14 mg group compared to the 3 mg group (p = 0.047). The proportion of participants transitioned from DPP-4 inhibitors was significantly higher in the 7 mg group compared to the 3 mg group (p < 0.001). These categorical variables correspond to those with statistically significant differences in **Table 1**.

Supplementary Figure 2 Box plots of hematological parameters stratified by oral semaglutide dose groups. **(A)** Red blood cell count (RBC). **(B)** Hemoglobin (Hb). **(C)** Hematocrit (Ht). Boxes represent the interquartile range (IQR), with the horizontal line inside each box indicating the median. Whiskers extend to 1.5 × IQR. *Post-hoc* comparisons were performed using the Bonferroni correction following Kruskal-Wallis tests. Significant differences were observed between the 3 mg and 14 mg groups for RBC (p = 0.014), Hb (p = 0.027), and Ht (p = 0.017). These continuous variables correspond to those with statistically significant differences in **Table 1**.
