# Supplementary material for: The impact of oral semaglutide on glycemic control and weight reduction: a database analysis of dosing effects in Japanese individuals with type 2 diabetes
Source: Front Endocrinol (Lausanne). 2025 Aug 26;16:1615516. doi: 10.3389/fendo.2025.1615516 (PMC12417148; doi:10.3389/fendo.2025.1615516)
Supplement: Supplementary Figure 1 — Bar charts of categorical baseline characteristics with significant differences across oral semaglutide dose groups. (A) Proportion of male participants. (B) Proportion of participants with prior use of DPP-4 inhibitors. A significantly higher proportion of males was observed in the 14 mg group compared to the 3 mg group (p = 0.047). The proportion of participants transitioned from DPP-4 inhibitors was significantly higher in the 7 mg group compared to the 3 mg group (p < 0.001). These categorical variables correspond to those with statistically significant differences in Table 1 . [file Presentation1.pptx]

## Slide 1
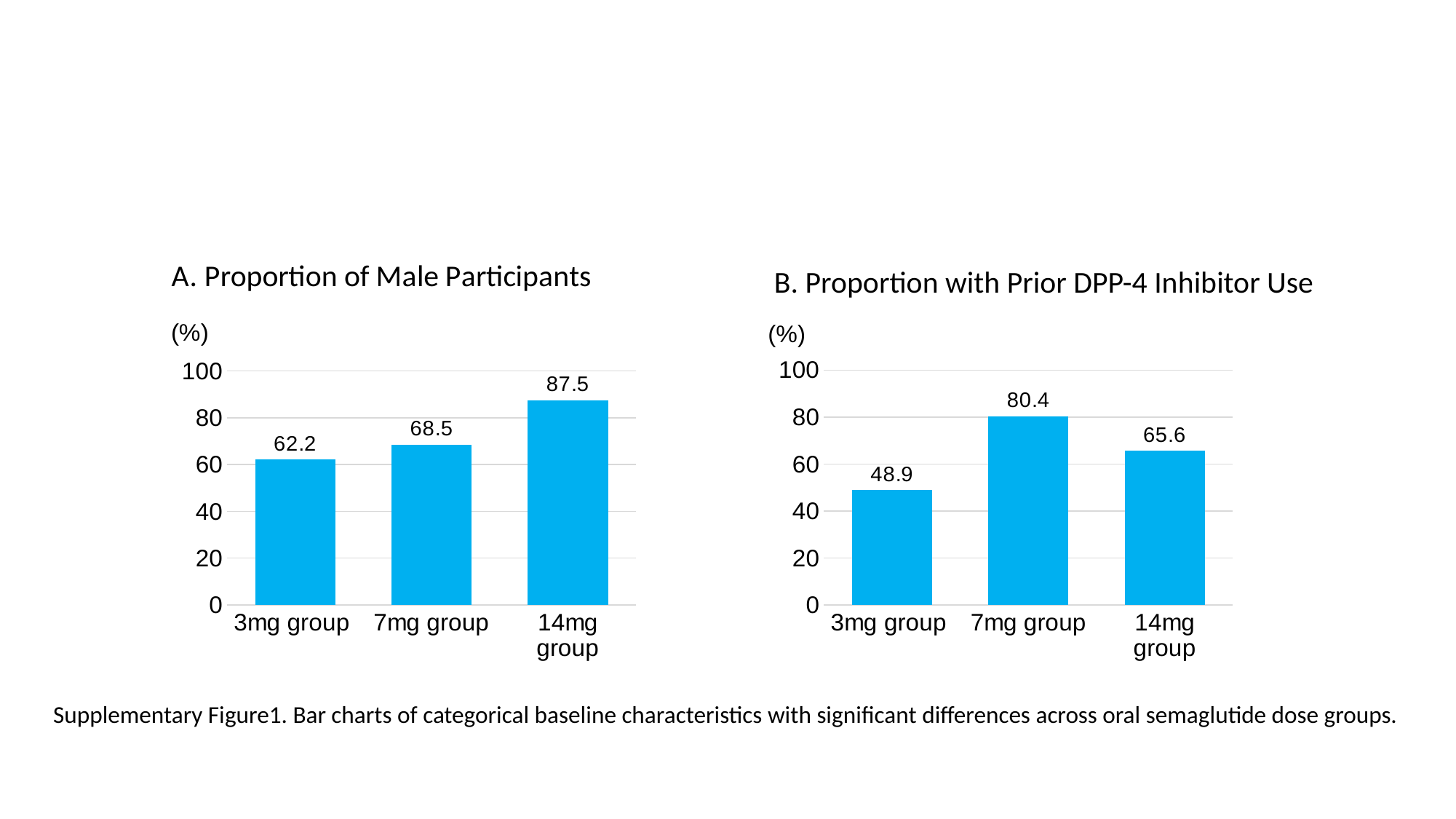

### Chart
| Category | |
|---|---|
| 3mg group | 62.2 |
| 7mg group | 68.5 |
| 14mg group | 87.5 |B. Proportion with Prior DPP-4 Inhibitor Use
### Chart
| Category | |
|---|---|
| 3mg group | 48.9 |
| 7mg group | 80.4 |
| 14mg group | 65.6 |Supplementary Figure1. Bar charts of categorical baseline characteristics with significant differences across oral semaglutide dose groups.

## Slide 2
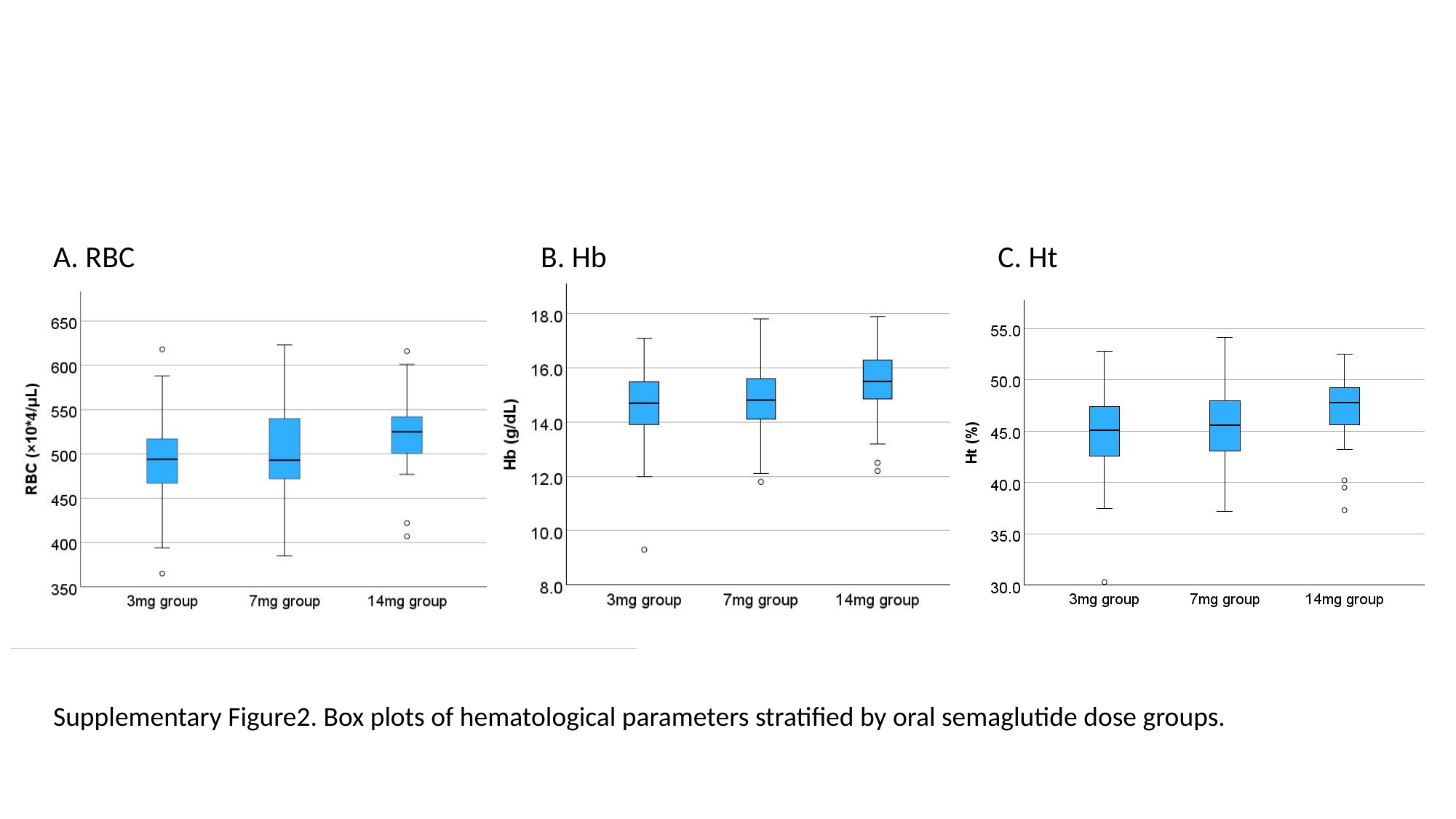

C. Ht
A. RBC
B. Hb
Supplementary Figure2. Box plots of hematological parameters stratified by oral semaglutide dose groups.
